# Clinical utility of the modified Glasgow prognostic score in lung cancer: A meta-analysis

**DOI:** 10.1371/journal.pone.0184412

**Published:** 2017-09-08

**Authors:** Jing Jin, Kejia Hu, Yongzhao Zhou, Weimin Li

**Affiliations:** 1 Department of Pulmonary & Critical Care, West China Hospital, Sichuan University, Chengdu, China; 2 Cancer Center, West China Hospital, Sichuan University, Chengdu, China; Taipei Medical University, TAIWAN

## Abstract

**Objective:**

To perform a meta-analysis of prospective and retrospective studies exploring the association of the modified Glasgow prognostic score (mGPS) with overall survival (OS) in patients with lung cancer.

**Methods:**

Relevant studies were identified by searching the Cochrane Library, Web of Science, Embase and PubMed until April 16, 2017. We combined hazard ratios (HRs) and 95% confidence intervals (CIs) to assess the correlation between mGPS and OS in patients with lung cancer.

**Results:**

Eleven studies involving 5817 participants from several countries were included in the meta-analysis. In a pooled analysis of all studies, elevated mGPS predicted poorer OS (HR = 1.77; 95% CI: 1.35–2.31; P<0.05). Subgroup analyses stratified by mGPS showed that mGPS of 1 or 2 and mGPS≥1 were predictive of poorer OS and that the HR for mGPS of 2 (HR = 5.82; 95% CI: 1.85–18.22; P = 0.003) was significantly greater than that for mGPS of 1 (HR = 1.74; 95% CI: 1.24–2.45; P = 0.001) and mGPS≥1 (HR = 1.42; 95% CI: 1.14–1.76; P = 0.002). Among patients undergoing surgery, elevated mGPS had a non-significant correlation with reduced OS (HR = 2.48; 95% CI: 0.90–6.85; P = 0.079), whereas the correlation was significant for patients receiving chemotherapy or other palliative treatment (HR = 1.74; 95% CI: 1.31–2.30; P<0.05).

**Conclusions:**

Our findings indicate that mGPS may have prognostic value in lung cancer, as we detected a significant association between elevated mGPS and poorer OS. The association between mGPS and poorer OS was non-significant among patients undergoing surgery, which may be attributable to lower tumor load. However, further studies are warranted to draw firm conclusions.

## Introduction

Lung cancer is one of the most common cancers and is the leading cause of all cancer mortality [1].

The primary treatments for lung cancer are surgery and chemotherapy [2]. With the development of earlier detection through computed tomography, the mortality rate of lung cancer has been reduced by 16%-20% among adults with a smoking history [3]. Although treatment and detection of lung cancer have improved and the 5-year survival rate has improved in recent years, an ideal method to predict the prognosis of lung cancer patients remains unavailable.

Mounting evidence has supported that systemic inflammation is related to shorter survival among patients with many cancers through promotion of cancer cell proliferation and survival, angiogenesis, and tumor metastasis [4, 5]. Inflammation-based prognostic scores including platelet-to-lymphocyte ratio (PLR), neutrophil-lymphocyte ratio (NLR), Glasgow Prognostic Score (GPS) and mGPS have been reported to have prognostic efficacy for patients with cancers [6, 7]. mGPS is a useful prognostic score for lung cancer. This score is based on C-reactive protein (CRP) and albumin, which represent not only inflammatory status but also nutritional status. Briefly, patients with both elevated CRP (>10 mg/L) and hypoalbuminemia (<35 g/L) are given mGPS of 2. Patients with serum CRP <10 mg/L with or without hypoalbuminemia receive scores of 0. Patients with only elevated CRP levels receive mGPS of 1.

Although accumulated evidence has demonstrated the clinical utility of mGPS in patients with different stages of lung cancer [8–17], data remain scarce and inconsistent. Therefore, we collected available publications and conducted a meta-analysis to explore the prognostic value of mGPS for OS in lung cancer.

## Methods

### Search strategy

We searched PubMed, the Cochrane Library, Web of Science and Embase for studies examining associations between mGPS and OS in patients with lung cancer published before April 2017. “Pulmonary Neoplasms”, “lung carcinoma”, “the modified Glasgow prognostic score”, “C-Reactive Protein” and “Albumin, Serum” were used separately or in combination for searching. Moreover, we explored the references cited in the selected articles and relevant reviews for additional publications. The publication language was limited to English, and there were no restrictions on the minimum number of patients. We screened titles and abstracts to identify related studies, and full texts were evaluated carefully. This meta-analysis was registered in PROSPERO (http://www.crd.york.ac.uk/PROSPERO) and the registration number for this article is CRD42017064263.

### Eligibility criteria

The inclusion criteria were as follows: 1) patients were diagnosed with lung cancer through biopsy; 2) studies were retrospective or prospective and published before April 2017; 3) mGPS was evaluated using C-reactive protein and serum albumin levels; 4) HRs and 95% CIs for mGPS and OS or data necessary to calculate them were reported in full text and published in English.

Exclusion criteria included: 1) nonhuman studies; 2) reviews, meeting abstracts and letters without full text in English; 3) studies which did not present mGPS values.

All identified studies were independently reviewed by two authors (J Jin and K Hu) for eligibility.

### Data extraction

Data were extracted by two researchers (J Jin and K Hu) independently. Any disagreements between them were resolved by discussion and consensus. The following information was recorded from all 11 studies: first author of the study, publication year, country of origin, study design, sample size and HR with 95% CI.

### Quality assessment

The primary studies were assessed using the NOS (Newcastle-Ottawa quality assessment scale). Studies which earned between 6 and 9 points were regarded as high-quality studies. Quality assessment was conducted by two independent researchers (J Jin and K Hu). (http://www.ohri.ca/programs/clinical_epidemiology/oxford.asp)

### Statistical analyses

We calculated the pooled HRs from each study in multivariate models whenever available using a random-effects model, which considers heterogeneity both between and within studies. HRs with 95% CIs were either directly obtained from the articles or estimated from the K-M curves according to the methods reported by Tierney et al [18]. For each study, the HRs comparing non-zero with zero mGPS values were then displayed in a forest plot. Statistical heterogeneity between studies was evaluated using Cochran’s Q test, with significant heterogeneity defined as P<0.10 [19]. Heterogeneity between studies was considered to be moderate or high if the I^2^ statistic was greater than 50%. Publication bias was evaluated using Begg’s test with significant publication bias defined as P<0.10 [20].

Sensitivity analyses were performed to rule out over-representation of results from a single study in the meta-analysis by excluding each study individually from the meta-analysis [21]. All statistical analyses were performed using STATA (version 12.0; Stata Corporation).

## Results

### Characteristics of included studies

**Fig 1:**
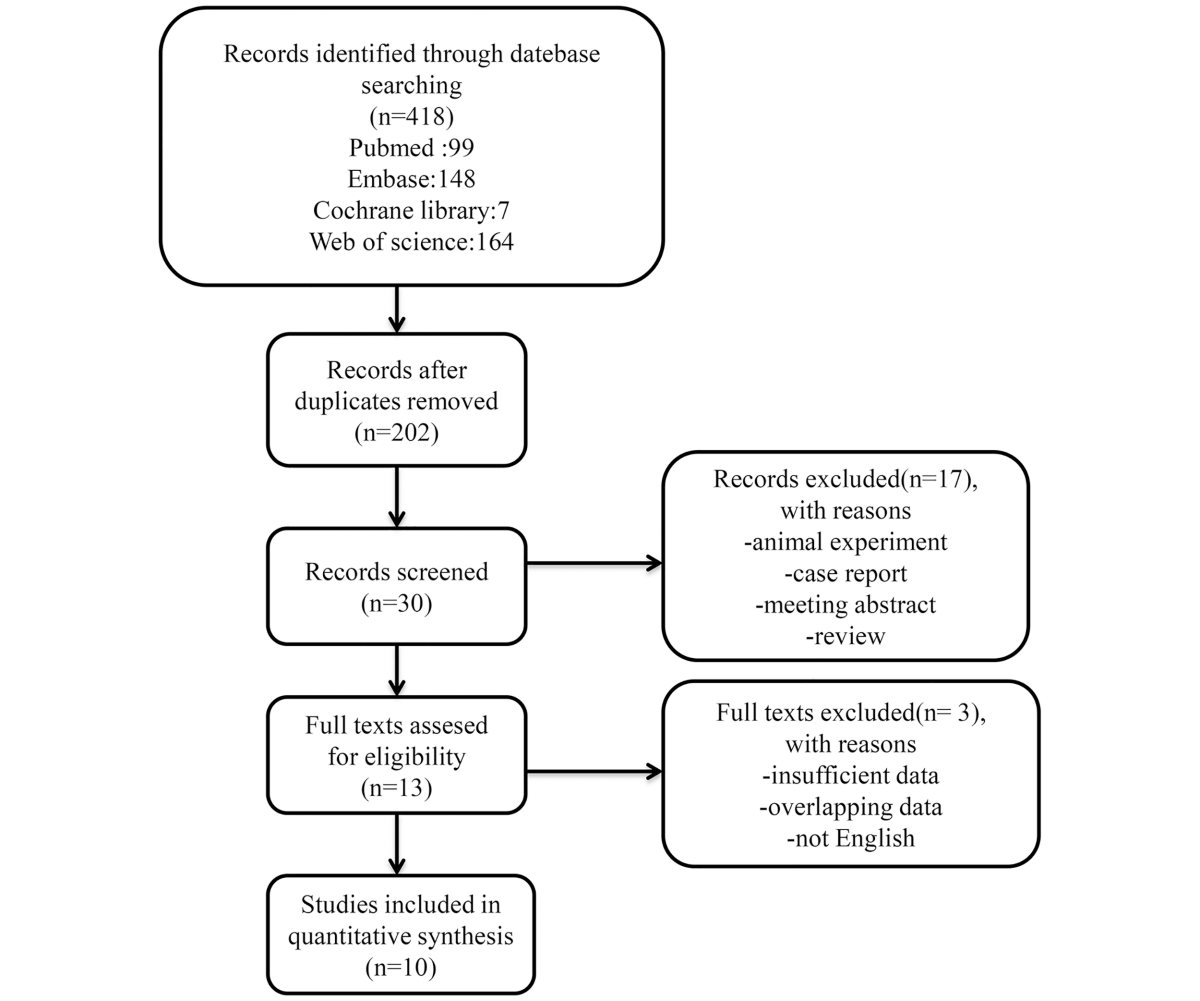
Flow chart of study selection.

The flow diagram of this study is presented in Fig 1. A total of 10 publications with 5817 patients were included in the meta-analysis [8–17]. One publication included two different cohorts (operative, inoperative) and reported their HRs separately, so we treated it as two studies [13]. The sample sizes ranged from 64 to 2988. Seven of the included publications were retrospective studies [12–17], and the remainder were prospective.

Of these studies, five studies were conducted in Europe and America [8–11, 15]. The remaining studies were conducted in Asia. 1983 patients from three studies [13, 16, 17] underwent surgery for lung cancer while the other patients received chemotherapy or other palliative treatment. Three studies included patients with small cell lung cancer (SCLC) [8, 10, 14] and seven studies [9, 11–13, 15–17] included patients with non-small cell lung cancer (NSCLC). HRs were not reported in three studies [8, 10, 14]; these were calculated from the respective K-M curves using the method mentioned above. With a reference score of 0, seven studies [9–13, 15, 16] provided the HRs for scores of 1 and 2. Finally, in this meta-analysis of 11 studies, the HR for lung cancer was calculated for the highest versus the lowest mGPS category. Almost all studies defined OS as the time between diagnosis and the date of death or last follow-up. The articles were published between 2012 and 2016 and the NOS scores of the included studies ranged from 6–9. Detailed information is shown in Table 1.

**Table 1:** Characteristics of all included studies.

Study	Year	Location	Ethnicity	Follow-up (months)	Sample size	Gender (M/F)	TNM stage	Treatment	Outcome	HR	Study design	NOS
**Zhu^12^**	2016	China	Asian	28.5(median)	105	72/33	IIIB-IV	chemotherapy	OS	R	RO	8
**Osugi^17^**	2016	Japan	Asian	65(median)	327	199/128	I-III	surgery	OS	R	RO	6
**Fan^13^**	2016	China	Asian	19(median)	1243	713/530	I-IV	surgery	OS	R	RO	8
**Fan^13^**	2016	China	Asian	20(median)	1745	1217/528	I-IV	chemotherapy	OS	R	RO	8
**Zhou^14^**	2015	China	Asian	NA	359	304/55	I-IV	chemotherapy	OS	E	RO	6
**Simmons^10^**	2015	UK	European	12.8(median)	390	341/49	IV	non-operative treatment	OS	E	PO	9
**Kishi^16^**	2015	Japan	Asian	42(median)	165	120/45	I	SBRT	OS	R	RO	7
**Pinato^9^**	2014	UK	European	NA	220	110/110	IA-IIIA	surgery	OS	R	PO	9
**Grose^8^**	2014	UK	European	24.5(median)	882	487/395	I-IV	mix	OS	E	PO	9
**Leung^11^**	2012	UK	European	83.1(median)	261	154/107	III-IV	active*/palliative care	OS	R	PO	9
**Meek^15^**	2012	UK	European	54(median)	56	34/22	II-IV	non-operative treatment	OS	R	RO	8

Abbreviation: HR: hazard ratio; OS: overall survival; PFS: progression-free survival; R: reported in article; E: estimated from K-M curves; PO: prospective studies; RO: retrospective studies; NOS: Newcastle-Ottawa quality assessment scale; SBRT: stereotactic body radiation therapy;

*: Patients were considered to have undergone active treatment if they received chemotherapy (mainly platinum-based) and/or radical radiotherapy.

### Relationship between mGPS and OS in lung cancer

**Fig 2:**
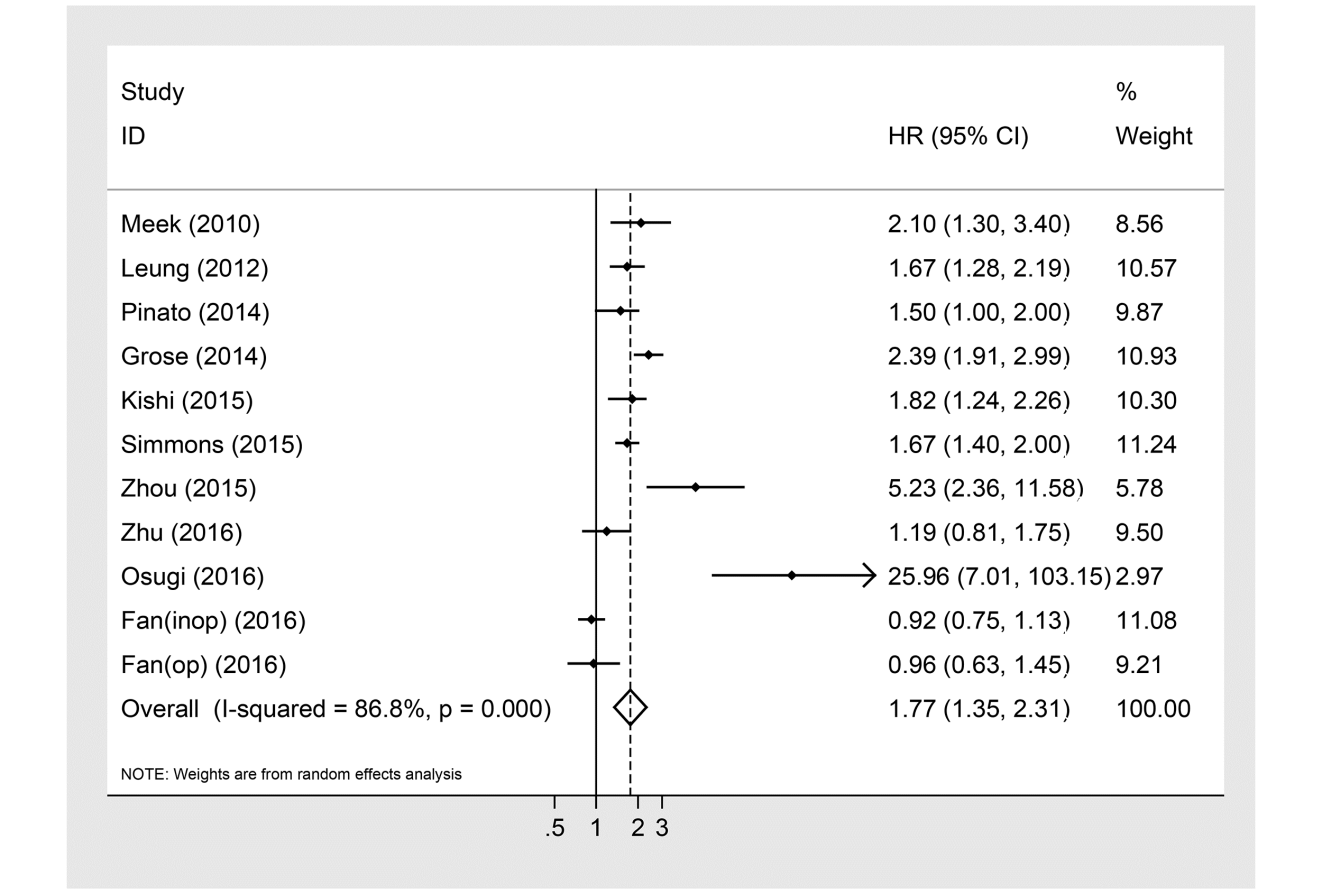
Forest plot of the association between mGPS and OS in patients with lung cancer.

Eleven studies with 5817 patients provided mGPS data before treatment and OS for patients with lung cancer. The random effects model revealed a significant relationship between elevated mGPS and OS in patients with lung cancer (HR: 1.77; 95% CI: 1.35–2.31; P<0.05) with high heterogeneity (I^2^ = 86.8%, p<0.001, Fig 2).

### Subgroup analyses

**Fig 3:**
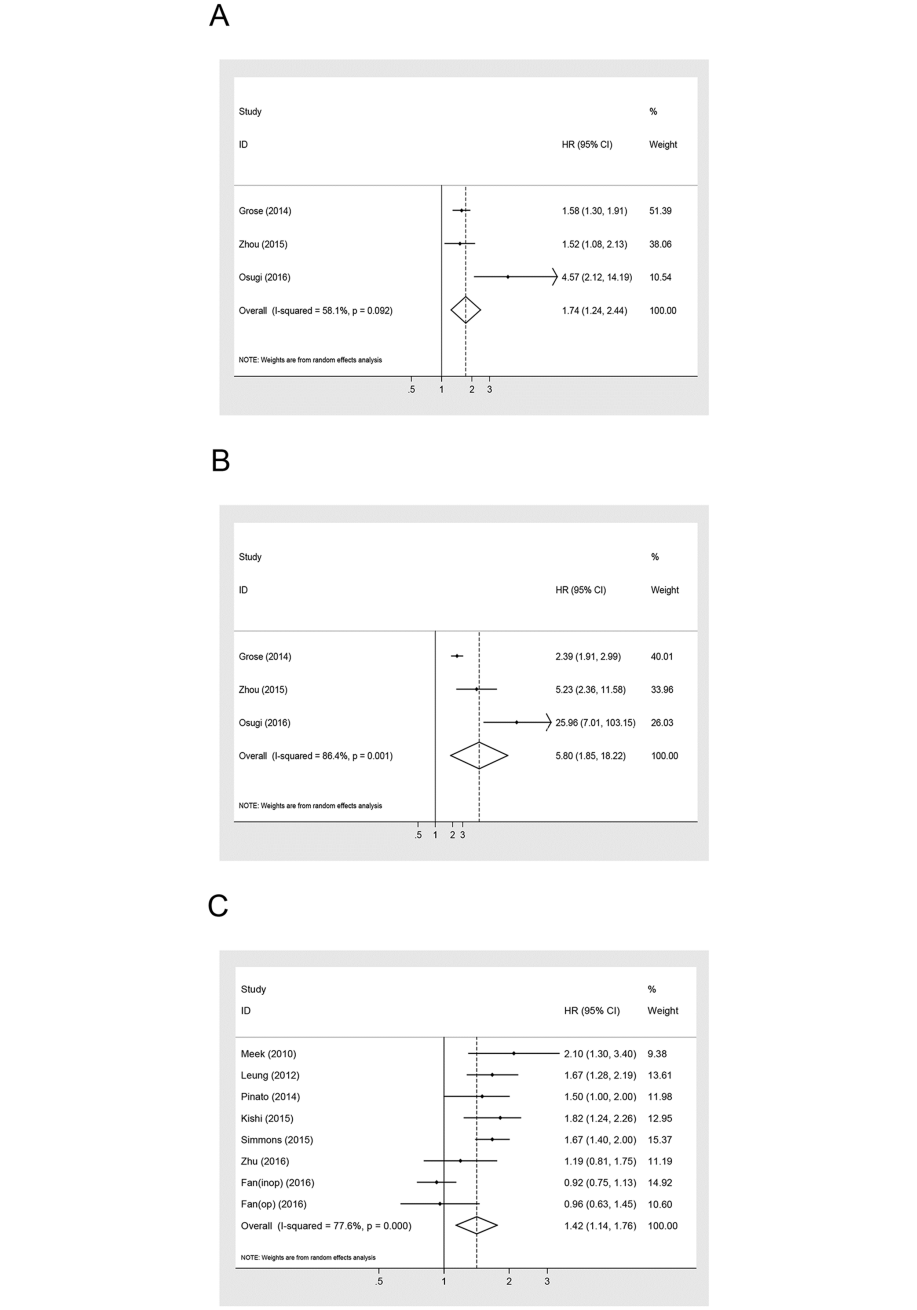
Forest plot of the association between mGPS and OS in patients with lung cancer stratified by pathology, ethnicity, treatment and study design.

**Fig 4:**
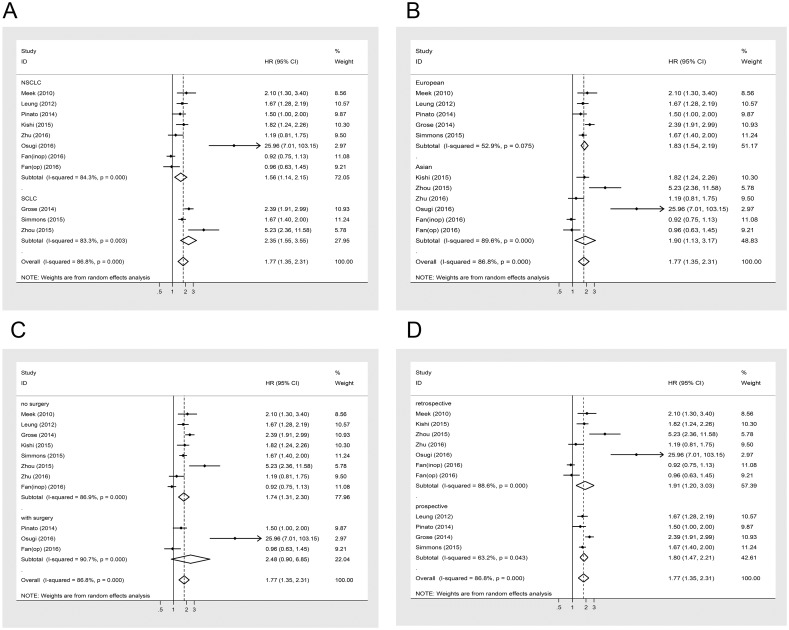
Forest plot of the association between mGPS and OS in patients with lung cancer stratified by mGPS.

Subgroup analyses stratified by mGPS, treatment, pathology subtypes, study design and ethnicity were conducted to detect potential sources of heterogeneity (Table 2, Figs 3 and 4). As shown in Table 2, mGPS of 1 or 2 predicted poor OS in patients with lung cancer (Fig 3A and 3B). Patients with mGPS ≥ 1 also had significantly poorer OS compared with those with mGPS = 0 (HR = 1.42; 95% CI: 1.14–1.76; P = 0.002) (I^2^ = 0%; P = 0.91) (Fig 3C). Moreover, compared with a reference score of 0, the HR for mGPS of 2 was significantly greater than for mGPS of 1 and ≥ 1 (Table 2). We also performed a subgroup analysis based on treatment to further explain the results of this meta-analysis. Among patients undergoing surgery, elevated mGPS had a non-significant correlation with shortened OS (HR = 2.48; 95% CI: 0.90–6.85; P = 0.079) (I2 = 86.8%; P<0.001) (Fig 4C). A significant correlation between mGPS and OS was observed among patients receiving chemotherapy or other palliative treatment (HR = 1.74; 95% CI: 1.31–2.30; P<0.05) (I^2^ = 86.9%; P<0.001). Regarding patients with different pathology subtypes, those with NSCLC (HR = 1.56; 95% CI: 1.14–2.15; P<0.05) and SCLC (HR = 2.35; 95% CI: 1.55–3.55; P<0.05) had similar trends in the relationship between mGPS and OS (Fig 4A). We also performed subgroup analyses of study design and ethnicity (Fig 4B and 4D). Significant relationships between increased mGPS and poorer OS were found with regard to different study designs and ethnicities (Table 2).

**Table 2:** Summary of HR for overall and subgroup analyses of mGPS and lung cancer.

		No. of studies	No. of participants	HR	95% CI	P	I^2^ (%)
**Overall**		11	5817	1.77	1.35–2.31	<0.001	86.8
**GPS**	OS						
	GPS = 1	3	415	1.74	1.24–2.44	0.001	58.1
	GPS = 2	3	232	5.80	1.85–18.22	0.003	86.4
	GPS≥1	8	4185	1.42	1.14–1.76	0.002	77.6
**Treatment**	Surgery	3	1983	2.48	0.90–6.85	0.079	90.7
	Chemotherapy	8	3834	1.74	1.31–2.30	<0.001	86.9
**Pathology**	NSCLC	8	4755	1.56	1.14–2.15	0.006	84.3
	SCLC	3	762	2.35	1.55–3.55	<0.001	83.3
**Study Design**	PO	4	1753	1.80	1.47–2.21	<0.001	63.2
	RO	7	4000	1.91	1.20–3.03	0.006	88.6
**Ethnicity**	European	5	1809	1.84	1.54–2.19	<0.001	52.9
	Asian	6	3944	1.90	1.13–3.17	0.015	89.6

### Sensitivity analysis and publication bias

**Fig 5:**
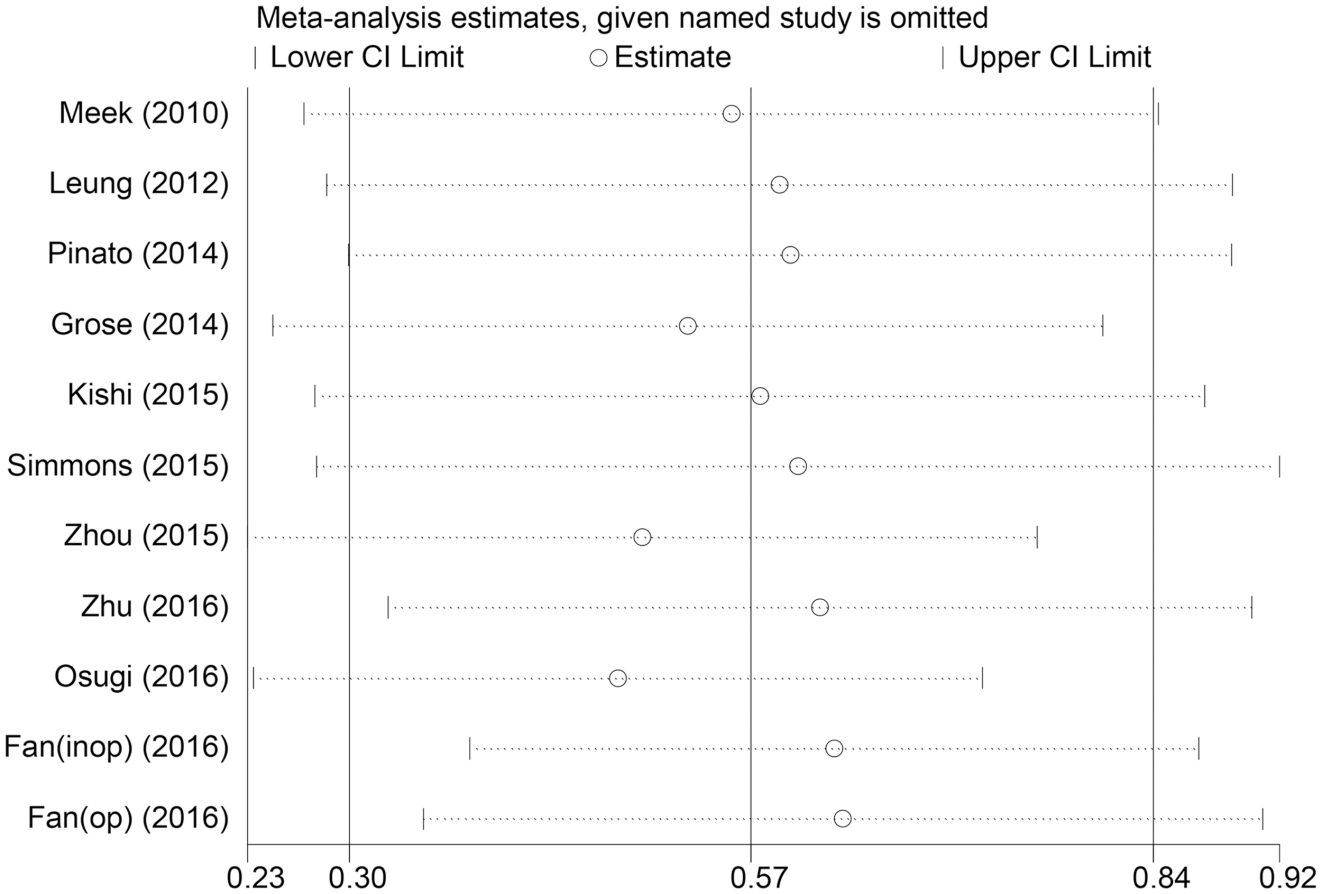
Sensitivity analysis of the relationship between mGPS and OS in lung cancer.

**Fig 6:**
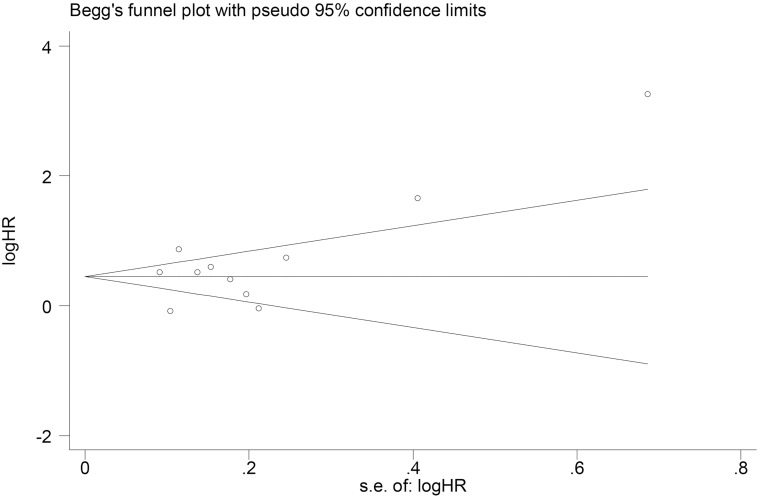
Begg’s funnel plot of publication bias testing for OS in lung cancer.

Significant heterogeneity was discovered among all studies (I^2^ = 86.6%, P<0.01), even after subgroup analyses. The influence of each single study set on the combined HRs was evaluated by excluding each study individually from the meta-analysis. The results showed that the pooled HRs for OS were robust in our study (Fig 5). Moreover, Begg’s test and the funnel plot showed no evidence of obvious publication bias (P = 0.350) (Fig 6).

## Discussion

The current meta-analysis summarizes the results of prospective and retrospective studies involving a total of 5817 patients. By combining the HRs and 95% CIs from 10 articles, we showed the prognostic impact of pretreatment mGPS on OS in patients with lung cancer. Our result revealed that elevated mGPS were significantly associated with poorer OS (HR: 1.77; 95% CI: 1.35–2.31; P<0.05). The subgroup analysis stratified by mGPS showed that the HR for mGPS of 2 was greater than for mGPS of 1 and ≥1. Furthermore, our results suggested that the association between elevated mGPS and poorer OS was non-significant in patients undergoing surgery (HR = 2.48; 95% CI: 0.90–6.85; P = 0.079), whereas high mGPS was significantly correlated with shortened OS in patients receiving chemotherapy or other palliative treatment (HR = 1.74; 95% CI: 1.31–2.30; P<0.05). Moreover, stratified analysis by pathology subtype showed that higher mGPS had consistent prognostic value in diverse subgroup populations and the HR for SCLC was greater than for NSCLC. To the best of our knowledge, this is the first meta-analysis to focus on the association between mGPS and lung cancer prognosis.

Systemic inflammation in patients with malignancy is considered to reflect the cytokine profile produced by the tumor [22]. Elevated levels of systemic inflammatory markers produced by solid tumors have been associated with worse survival [23] [24]. Other available blood-based biomarkers including NLR, PLR and mGPS could reflect the cancer-related inflammatory status and have been used as prognostic factors in lung cancer [7, 25–28]. As an important biomarker of systemic inflammation, C-reactive protein (CRP) is synthesized by liver cells in response to microbial invasion or tissue injury [29]. Its prognostic value has been investigated in many studies in patients with various cancer types [30–36]. Albumin can reflect the nutritional status of patients with cancers; malnutrition is correlated with worse survival [37]. A mGPS evaluating both systemic inflammation and nutritional status may serve as potential prognostic predictor for lung cancer, as has been assessed in many studies [8–17]. There are also several meta-analyses concerning mGPS and survival in other kinds of tumors such as gastric cancer [38], colorectal cancer [39] and hepatocellular carcinoma [40]. Zhang et al. [38] showed that OS was worse in gastric cancer patients with mGPS of 1 and 2 (OR = 2.54 (1.62, 3.98) and OR = 12.02 (6.79, 21.28), respectively) compared with scores of 0. Woo et al. [39] provided evidence that the pooled HR for OS of those with higher GPS/mGPS was 2.20 (95% CI 1.61–3.02) in earlier stage colorectal cancer surgery patients. Chen et al. [40] reported that the HR of mGPS for OS in patients with hepatocellular carcinoma was 2.21 (1.73–2.82). Liu et al. showed that for OS, the pooled HR of elevated CRP in prostate cancer patients was statistically significant at 1.51 (95% CI, 1.28–1.79) [41]. The HR or OR seemed to be slightly higher in these tumors. Additionally, HR was generally similar across different tumors. In summary, mGPS is a reliable prognostic factor for patients with various cancer types. The results of our study showed that elevated mGPS is a prognostic factor for OS in lung cancer, a finding consistent with most studies [8–12, 14–17]. Additionally, we observed that the HR for mGPS of 2 was significantly greater than for mGPS of 1 and ≥1, which may indicate that higher mGPS are related to poorer OS. For patients with high pretreatment mGPS, we should take a more positive approach and observe progression according to our results. mGPS may not only identify patients at risk but also may provide a well-defined therapeutic target for future clinical trials [42]. A recent meta-analysis [43] advocated interventions with nonsteroidal anti-inflammatory drugs (NSAIDs) due to their apparent ability to reduce risk of metastasis development, regardless of pre-diagnostic or post-diagnostic use.

However, due to the limited number of studies we were able to include, these results must be interpreted with caution. For patients with different treatment modalities, we found that OS for patients undergoing surgery was not significantly correlated with mGPS, which was inconsistent with the findings for patients receiving chemotherapy or other palliative treatment. The results for patients undergoing surgery were similar to those reported previously by Fan, H [13], although they were inconsistent with results presented by Osugi, J and Pinato, DJ [9, 17]. In our study, the HR for patients undergoing surgery was 2.48 and the P value was 0.079, which was slightly higher than the cutoff value (0.05). A possible explanation is that patients who were operable were usually at an earlier stage and thus suffering from lower tumor burdens. Moreover, the survival time for patients at earlier stages may be longer and more easily affected by other factors such as operative method, lifestyle, other biomarkers, etc. Finally, we observed that mGPS had prognostic value in diverse subgroup populations and that the HR for SCLC was greater than for NSCLC, which may suggest that systemic inflammatory response activity differed in patients with different pathology subtypes. This finding needs more prospective cohort studies for validation. Although most survival data were extracted from multivariate models adjusted for potential confounding factors including gender, age, stage, treatment and other biomarkers, a few studies only provided survival data based on univariable analysis. This factor may have been responsible for the high heterogeneity in this analysis. However, due to the limited information presented in the studies, it was not possible to perform subgroup analyses according to different cofounding factors.

The current research had several limitations. First, high heterogeneity was present in this meta-analysis. Although sensitivity analyses and publication bias testing indicated credibility of the results, we could not rule out the possibility that adjustments for cofounding factors or the primary study criteria resulted in discrepancies between studies. Second, participants from enrolled studies were at different clinical stages. 3594 patients with advanced stage cancers (III and IV) and 1987 patients with early stage cancers (Iand II) were involved in this meta-analysis, although we were not able to perform subgroup analyses according to different clinical stages. One final limitation in our study was that the cut off values for C-reactive protein and serum albumin were 0.3 mg/dL and 3.5 g/dL, respectively. These levels were different from other studies included in our meta-analysis which defined cut off values of 10 mg/L for CRP and 35 g/L for serum albumin. The conditions and instruments for measurement in different laboratories may also have affected the CRP and albumin thresholds.

Generally, our meta-analysis was the first to demonstrate the prognostic role of increased mGPS for poor OS, although the OS for patients undergoing surgery was not correlated with mGPS. Nonetheless, given the limitations mentioned above, these findings should be treated with caution in clinical practice. More prospective cohort studies are warranted to test our results.

## Supporting information

S1 File Search strategy. This file provides a full electronic search strategy for PubMed.
(DOCX)

S2 File Full-text excluded articles. This file lists the full-text excluded articles in a supporting information file, giving the reasons for exclusion.
(DOCX)

S3 File Data set. This file provides the minimal anonymized data set necessary to replicate our findings.
(XLSX)

S4 File PRISMA 2009 checklist. (DOC)

S5 File Meta-analysis protocol. This file provides the protocol for this meta-analysis.
(PDF)
