# Supplementary material for: Clinical utility of the modified Glasgow prognostic score in lung cancer: A meta-analysis
Source: PLoS One. 2017 Sep 8;12(9):e0184412. doi: 10.1371/journal.pone.0184412 (PMC5590927; doi:10.1371/journal.pone.0184412)
Supplement: S1 File — This file provides a full electronic search strategy for PubMed. (DOCX) [file pone.0184412.s001.docx]

**Table S1 Search strategy for meta-analysis of Clinical utility of the modified Glasgow prognostic score in lung cancer (PubMed via NLM)**

|  | **Search terms: *the modified Glasgow prognostic score and lung cancer*** | **Items found** |
| --- | --- | --- |
| ***Population: persons with lung cancer*** | | |
| 1 | ((((((((((((((((((Cancer of Lung) OR Pulmonary Neoplasms) OR Neoplasms, Lung) OR Lung Neoplasm) OR Neoplasm, Lung) OR Neoplasms, Pulmonary) OR Neoplasm, Pulmonary) OR Pulmonary Neoplasm) OR Lung Cancer) OR Cancer, Lung) OR Cancers, Lung) OR Lung Cancers) OR Pulmonary Cancer) OR Cancer, Pulmonary) OR Cancers, Pulmonary) OR Pulmonary Cancers) OR Cancer of the Lung) OR "Lung Neoplasms"[Mesh] | **315807** |
| ***Intervention (Expose): the modified Glasgow prognostic score*** | | |
| 2 | (((((("Serum Albumin"[Mesh]) OR Albumin, Serum) OR Plasma Albumin)))) AND (("C-Reactive Protein"[Mesh]) OR C Reactive Protein) OR Protein, C-Reactive)))) OR the modified Glasgow score | **64485** |
| ***Study types*** | | |
| 3 | (((Clinical Trial[Publication Type]) OR review[Publication Type]) OR cohort study[Publication Type]) OR case control study[Publication Type] | **3030071** |
| ***Combined sets*** | | |
| 4 | 1 and 2 and 3 | **103** |
| ***Limits*** | | |
| 5 | 4 AND English[Language] | **102** |

[Mesh] = Term from the Medline controlled vocabulary, including terms found below this term in the Mesh hierarchy
