# Supplementary material for: Clinical utility of the modified Glasgow prognostic score in lung cancer: A meta-analysis
Source: PLoS One. 2017 Sep 8;12(9):e0184412. doi: 10.1371/journal.pone.0184412 (PMC5590927; doi:10.1371/journal.pone.0184412)
Supplement: S2 File — This file lists the full-text excluded articles in a supporting information file, giving the reasons for exclusion. (DOCX) [file pone.0184412.s002.docx]

The full-text excluded articles:

1. Validation of the Modified Glasgow Prognostic Score in Advanced Cancer Patients Receiving Palliative Care (1)

The reason for exclusion: this article did not give insufficient data about survival time and mGPS in patient with lung cancer

2. The Glasgow inflammatory score and lung cancer: A predictor of admissions to emergency units (2)

The reason for exclusion: this article was not in English

3. An inflammation-based prognostic score (mGPS) predicts cancer survival independent of tumor site: a Glasgow Inflammation Outcome Study (3)

The reason for exclusion: This article provided HRs of mGPS for OS in several kinds of cancer, including lung cancer. But lung cancer is not an independent disease of interest in this article and it did not provide sufficient data for patients with lung cancer to calculate the HRs of mGPS for OS in patients with lung cancer.

1. Pantano Nde P, Paiva BS, Hui D, Paiva CE. Validation of the Modified Glasgow Prognostic Score in Advanced Cancer Patients Receiving Palliative Care. Journal of pain and symptom management. 2016;51(2):270-7.

2. Gorham J, Ameye L, Paesmans M, Berghmans T, Sculier JP, Meert AP. [The Glasgow inflammatory score and lung cancer: A predictor of admissions to emergency units]. Revue des maladies respiratoires. 2016;33(9):759-65.

3. Proctor MJ, Morrison DS, Talwar D, Balmer SM, O'Reilly DS, Foulis AK, et al. An inflammation-based prognostic score (mGPS) predicts cancer survival independent of tumour site: a Glasgow Inflammation Outcome Study. British journal of cancer. 2011;104(4):726-34.
