# Supplementary material for: Clinical utility of the modified Glasgow prognostic score in lung cancer: A meta-analysis
Source: PLoS One. 2017 Sep 8;12(9):e0184412. doi: 10.1371/journal.pone.0184412 (PMC5590927; doi:10.1371/journal.pone.0184412)
Supplement: S5 File — This file provides the protocol for this meta-analysis. (PDF) [file pone.0184412.s005.pdf]

PROSPERO International prospective register of systematic reviews

Review title and timescale

1 Review title
Give the working title of the review. This must be in English. Ideally it should state succinctly the interventions or exposures being reviewed and the associated health or social problem being addressed in the review.
Prognostic value of the modified Glasgow prognostic score in lung cancer: a meta-analysis

2 Original language title
For reviews in languages other than English, this field should be used to enter the title in the language of the review. This will be displayed together with the English language title.

3 Anticipated or actual start date
Give the date when the systematic review commenced, or is expected to commence.
01/04/2017

4 Anticipated completion date
Give the date by which the review is expected to be completed.
31/05/2017

5 Stage of review at time of this submission
Indicate the stage of progress of the review by ticking the relevant boxes. Reviews that have progressed beyond the point of completing data extraction at the time of initial registration are not eligible for inclusion in PROSPERO. This field should be updated when any amendments are made to a published record.

The review has not yet started ×

Review stage	Started	Completed
Preliminary searches	No	Yes
Piloting of the study selection process	No	Yes
Formal screening of search results against eligibility criteria	No	Yes
Data extraction	Yes	No
Risk of bias (quality) assessment	Yes	No
Data analysis	Yes	No

Provide any other relevant information about the stage of the review here.

Review team details

6 Named contact
The named contact acts as the guarantor for the accuracy of the information presented in the register record.
Dr Hu

7 Named contact email
Enter the electronic mail address of the named contact.
janey7966@163.com

8 Named contact address
Enter the full postal address for the named contact.
Sichuan University, Chengdu, China

9 Named contact phone number
Enter the telephone number for the named contact, including international dialing code.
+86 18200524467

10 Organisational affiliation of the review
Full title of the organisational affiliations for this review, and website address if available. This field may be completed as 'None' if the review is not affiliated to any organisation.
Sichuan University

Website address:

11 Review team members and their organisational affiliations

Give the title, first name and last name of all members of the team working directly on the review. Give the organisational affiliations of each member of the review team.

Title	First name	Last name	Affiliation
Dr	Jing	Jin	Department of Pulmonary & Critical Care, West China Hospital, Sichuan University, Chengdu 610041, China
Dr	Kejia	Hu	Cancer Center, West China Hospital, Sichuan University, Chengdu 610041, China
Dr	Yong-Zhao	Zhou	Department of Pulmonary & Critical Care, West China Hospital, Sichuan University, Chengdu 610041, China.
Professor	Wei-Min	Li	Department of Pulmonary & Critical Care, West China Hospital, Sichuan University, Chengdu 610041, China.

12 Funding sources/sponsors

Give details of the individuals, organizations, groups or other legal entities who take responsibility for initiating, managing, sponsoring and/or financing the review. Any unique identification numbers assigned to the review by the individuals or bodies listed should be included.

None.

13 Conflicts of interest

List any conditions that could lead to actual or perceived undue influence on judgements concerning the main topic investigated in the review.

Are there any actual or potential conflicts of interest?

None known

14 Collaborators

Give the name, affiliation and role of any individuals or organisations who are working on the review but who are not listed as review team members.

Title	First name	Last name	Organisation details

Review methods

15 Review question(s)

State the question(s) to be addressed / review objectives. Please complete a separate box for each question.

Does the modified Glasgow prognostic score plays a prognostic role in lung cancer?

16 Searches

Give details of the sources to be searched, and any restrictions (e.g. language or publication period). The full search strategy is not required, but may be supplied as a link or attachment.

The databases of Cochrane Library, Web of Science, Embase and PubMed will be searched. The following key words will be used in separation or in combination: “Pulmonary Neoplasms”, “lung carcinoma”, “Pulmonary Cancer”, “C-Reactive Protein”, “Albumin, Serum” and “modified Glasgow prognostic score”. The publication language will be limited to English.

17 URL to search strategy

If you have one, give the link to your search strategy here. Alternatively you can e-mail this to PROSPERO and we will store and link to it.

I give permission for this file to be made publicly available

Yes

18 Condition or domain being studied

Give a short description of the disease, condition or healthcare domain being studied. This could include health and wellbeing outcomes.
Lung cancer is one of the most common cancers and the leading cause of all cancers. The main treatments for lung cancer were surgery and chemotherapy. With the development of earlier detection through computed tomography, the death rate of lung cancer reduced 16%-20% in adults with smoking history. Although the treatments and detection in lung cancer have improved and the 5 year survival rate has decreased in recent years, the ideal method to predict the prognosis of lung cancer remains unavailable.

19 Participants/population
Give summary criteria for the participants or populations being studied by the review. The preferred format includes details of both inclusion and exclusion criteria.
The following inclusion criteria will be used: patients were pathologically diagnosed as lung cancer. The exclusion criteria will be as follows: nonhuman studies.

20 Intervention(s), exposure(s)
Give full and clear descriptions of the nature of the interventions or the exposures to be reviewed
The following inclusion criteria will be used: mGPS was measured based on C-reactive protein and albumin of serum; provision of HRs and 95% CIs for mGPS in OS or data necessary to calculate them. The exclusion criteria will be as follows: did not present the value for mGPS.

21 Comparator(s)/control
Where relevant, give details of the alternatives against which the main subject/topic of the review will be compared (e.g. another intervention or a non-exposed control group).
The control group comprises those with a mGPS of 0.

22 Types of study to be included
Give details of the study designs to be included in the review. If there are no restrictions on the types of study design eligible for inclusion, this should be stated.
The following inclusion criteria will be used: retrospective or prospective study design. The exclusion criteria will be as follows: review, meeting abstract, and letter, no full text in English.

23 Context
Give summary details of the setting and other relevant characteristics which help define the inclusion or exclusion criteria.

24 Primary outcome(s)
Give the most important outcomes.
Overall survival

Give information on timing and effect measures, as appropriate.

25 Secondary outcomes
List any additional outcomes that will be addressed. If there are no secondary outcomes enter None.
None.

Give information on timing and effect measures, as appropriate.

26 Data extraction (selection and coding)
Give the procedure for selecting studies for the review and extracting data, including the number of researchers involved and how discrepancies will be resolved. List the data to be extracted.

27 Risk of bias (quality) assessment
State whether and how risk of bias will be assessed, how the quality of individual studies will be assessed, and whether and how this will influence the planned synthesis.
The quality assessment of primary studies will be performed according to Newcastle-Ottawa quality assessment Scale (NOS). The full mark is 9 points and studies labeled with more than 6 points will be regarded as high-quality researches.

28 Strategy for data synthesis
Give the planned general approach to be used, for example whether the data to be used will be aggregate or at the

level of individual participants, and whether a quantitative or narrative (descriptive) synthesis is planned. Where appropriate a brief outline of analytic approach should be given.
We will provide a narrative synthesis of the findings from the included studies, target population characteristics, different scores of mGPS and corresponding HRs and 95% CIs. We will provide summaries of intervention effects for each study by calculating pooled hazard ratios. We use the hazards ratios in multivariate models whenever available. If there are only univariate models presented, we will use the hazards ratios in univariate models to calculate the pooled HRs. We anticipate that there will be limited scope for meta-analysis because of the range of different outcomes measured across the small number of existing trials. However, where studies have given the same type of exposure and control group, with the same outcome measure, we will pool the results using a random-effects meta-analysis, with hazard ratios for OS, and calculate 95% confidence intervals and two sided P values for each outcome. Heterogeneity between the studies in effect measures will be assessed using both the Q test and the I-squared statistic. We will consider an I-squared value greater than 50% indicative of substantial heterogeneity. We will conduct sensitivity analyses based on study quality. We will use stratified meta-analyses to explore heterogeneity in effect estimates according to: treatment and different scores of mGPS. We will also assess evidence of publication bias.

29 Analysis of subgroups or subsets
Give any planned exploration of subgroups or subsets within the review. 'None planned' is a valid response if no subgroup analyses are planned.
Subgroup analyses stratified by different scores of mGPS will be conducted.

Review general information

30 Type and method of review
Select the type of review and the review method from the drop down list.
Meta-analysis, Systematic review

31 Language
Select the language(s) in which the review is being written and will be made available, from the drop down list. Use the control key to select more than one language.
English

Will a summary/abstract be made available in English?
Yes

32 Country
Select the country in which the review is being carried out from the drop down list. For multi-national collaborations select all the countries involved. Use the control key to select more than one country.
China

33 Other registration details
Give the name of any organisation where the systematic review title or protocol is registered together with any unique identification number assigned. If extracted data will be stored and made available through a repository such as the Systematic Review Data Repository (SRDR), details and a link should be included here.

34 Reference and/or URL for published protocol
Give the citation for the published protocol, if there is one.
Give the link to the published protocol, if there is one. This may be to an external site or to a protocol deposited with CRD in pdf format.

I give permission for this file to be made publicly available
Yes

35 Dissemination plans
Give brief details of plans for communicating essential messages from the review to the appropriate audiences.
Do you intend to publish the review on completion?
Yes

36 Keywords
Give words or phrases that best describe the review. (One word per box, create a new box for each term)

mGPS

systemic inflammation

prognosis

lung cancer

37 Details of any existing review of the same topic by the same authors
Give details of earlier versions of the systematic review if an update of an existing review is being registered, including full bibliographic reference if possible.

38 Current review status
Review status should be updated when the review is completed and when it is published.
Ongoing

39 Any additional information
Provide any further information the review team consider relevant to the registration of the review.

40 Details of final report/publication(s)
This field should be left empty until details of the completed review are available.
Give the full citation for the final report or publication of the systematic review.
Give the URL where available.
